# Supplementary material for: How to account for behavioral states in step-selection analysis: a model comparison
Source: PeerJ. 2024 Feb 26;12:e16509. doi: 10.7717/peerj.16509 (PMC10903358; doi:10.7717/peerj.16509)
Supplement: Supplemental Information 1 [file peerj-12-16509-s001.pdf]

Supplementary Material for: "How to account for behavioral states in step selection analysis: a model comparison"

J. Pohle[1]*, J. Signer[2], J. A. Eccard[3], M. Dammhahn[4] and U. E. Schlägel[1]

[1]Institute of Biochemistry and Biology, University of Potsdam, Potsdam, Germany
[2]Wildlife Sciences; Faculty of Forest Sciences and Forest Ecology; University of Goettingen, Göttingen, Germany
[3]Animal Ecology, Institute of Biochemistry and Biolog, University of Potsdam, Potsdam, Germany
[4] Department of Behavioural Biology, University of Münster, Münster, Germany

*Corresponding author; email: jennifer.pohle@uni-potsdam.de; postal address: Am Mühlenberg 3, building 60, 14476 Potsdam, Germany.

Contents

S1 Illustration of state-dependent step-selection densities **3**

S2 Movement covariates and sampling procedures **4**

S2.1 Sampling procedures for the control steps 4

S2.2 Movement parameters and corresponding movement covariates 5

S3 Initial values for the maximum likelihood estimation **7**

S4 Supplementary material for the simulation study **9**

S4.1 Simulated landscape feature 9

S4.2 Bias 10

S4.3 Root mean squared error 11

S5 Additional simulation runs **12**

S5.1 Uniform sampling of the control locations 12

S5.2 Scenario without state-switching 13

S5.3 Habitat feature with less spatial variation 16

S6 Supplementary material for the bank vole case study **19**

S6.1 Animal care approval and research permissions 19

S6.2 Selection coefficient estimates for different numbers of control steps 20

S6.3 HMM-iSSA estimates for interactions with same-sex conspecifics 21

S6.4 TS-iSSA results 22

S6.5 Viterbi-decoding 25

S6.6 Information criteria results 26

References **27**

S1 Illustration of state-dependent step-selection densities

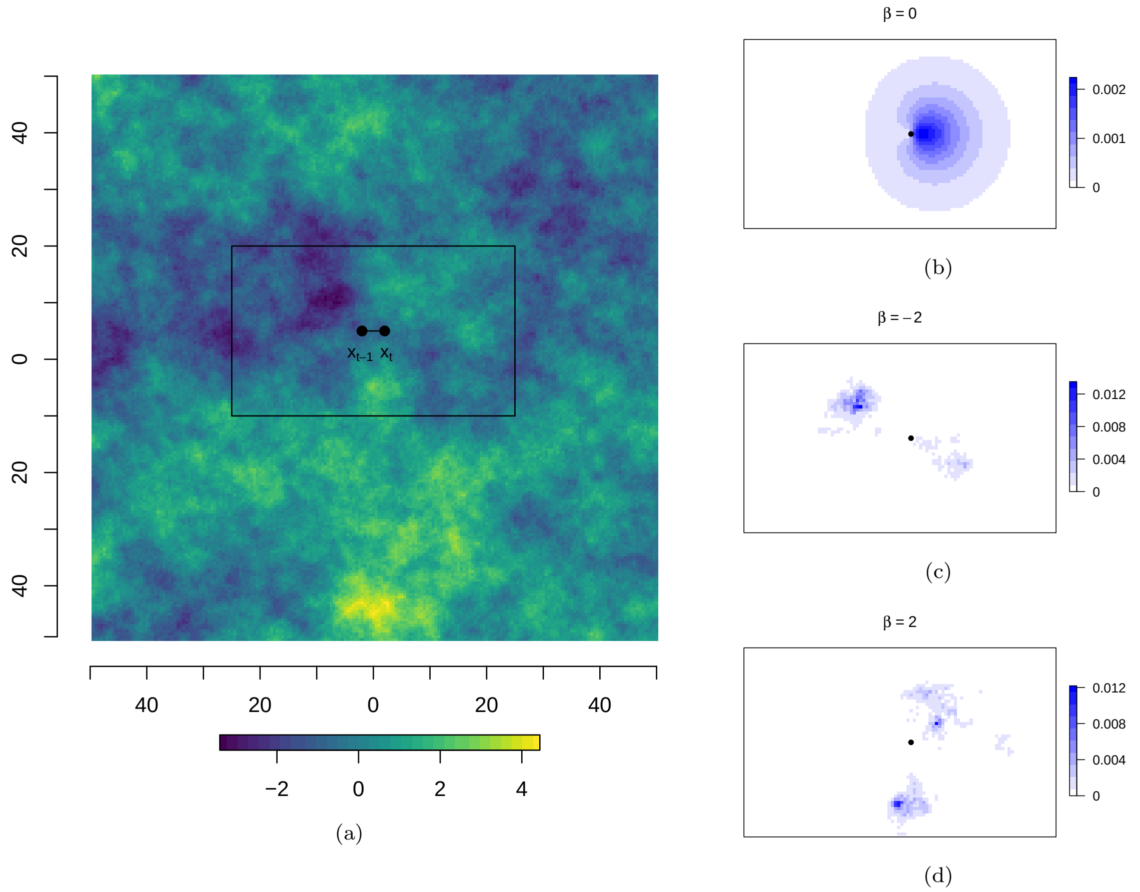

Figure S1: Illustration of the state-dependent step selection density f_i in a 2-state setting as used in Scenario 2 of the simulation study (Section 2.4 main manuscript). Panel a) shows a part of the simulated landscape feature map. The two black dots exemplify the past and current location of the animal. Panel b) shows the movement kernel which consists of a gamma distribution for step length and a von Mises distribution with mean zero for turning angle. In this example, the movement kernel is the same for both states. Panel b) and c) depict the step-selection density for state 1 (f_1) and state 2 (f_2), respectively. In state 1, the selection coefficient is set to $\beta_1 = -2$ which corresponds to avoidance of the landscape feature, while in state 2, the animal is attracted to the feature ($\beta_2 = 2$).

S2 Movement covariates and sampling procedures

S2.1 Sampling procedures for the control steps

There are different possibilities to sample the control, i.e. available steps for the iSSA or HMM-iSSA. For iSSAs, usually a procedure related to importance sampling is used which can be described as follows (Avgar et al., 2016):

1) Proposal distribution:

   1a) Fit the distribution d_l chosen to model step length (e.g. gamma distribution) to the observed step lengths $l_{0,t}$ $(t = 2, \ldots, T)$ to obtain the proposal distribution $d_{l,\text{prop}}$ with corresponding parameter vector $\boldsymbol{\theta}_{l,\text{prop}}$ (e.g. containing the shape k_{prop} and rate r_{prop} of the fitted gamma distribution).

   1b) If a von Mises distribution with fixed mean is assumed for turning angle, fit the corresponding von Mises distribution d_α to the observed turning angles $\alpha_{0,t}$ $(t = 3, \ldots, T)$ to obtain the proposal distribution $d_{\alpha,\text{prop}}$ with concentration parameter κ_{prop}.

2) Random steps and locations:

   2a) For each step starting at $t = 2, \ldots, T-1$, draw M random step length $l_{m,t+1}$ from the proposal distribution $d_{l,\text{prop}}$.

   2b) For each step starting at $t = 2, \ldots, T-1$, further draw M random turning angles $\alpha_{m,t+1}$ either from the proposal distribution $d_{\alpha,\text{prop}}$ if a von Mises distribution is assumed or from a uniform distribution with boundaries $-\pi$ and π.

   2c) Use the M randomly drawn step lengths and turning angles together with the starting point $\mathbf{x}_{0,t}$ to compute the M control locations $\mathbf{x}_{m,t+1}$ and their corresponding movement covariates $\mathbf{C}_{m,t+1}$ $(m = 1, \ldots, M)$.

3) Extract the habitat covariates $\mathbf{Z}_{m,t+1}$ for each $\mathbf{x}_{m,t+1}$ from $\mathbf{Z}$ $(m = 1, \ldots, M,\ t = 2, \ldots, T-1)$.

Another possibility to sample the control steps is the use of a uniform distribution for both the step length and turning angle of each control step. For the turning angles, values between $-\pi$ and π are a natural choice. For step length, however, an upper limit must be chosen. One possibility is to add a constant value, e.g. 10, to the maximum observed step length. Another possibility is to use the 99.9%-quantile of the proposal distribution $d_{l,\text{prop}}$ described in step 1a) of the importance sampling procedure described above. Note that the upper step length value must be chosen large enough to cover the main support of the estimated state-dependent step length distributions, otherwise it might lead to an estimation bias (Nicosia et al., 2017).

It is possible to combine both sampling approaches. For the simulation study described in Section 2.4 (main manuscript), we drew the random step lengths from a gamma proposal distribution, while the random turning angles were sampled from a uniform distribution (although we assumed a von Mises distribution for turning angles).

Furthermore, it is possible to lay a grid or a mesh either over the complete animals' domain or over a buffer area around the current animal location (Schlägel and Lewis, 2014; Arce Guillen et al., 2023). For a grid, the mid-points of each grid cell provide the end-point locations for each control step. A fine resolution of the grid improves the estimation accuracy, but might come with high computational

costs. Thus, it is important to find a good balance between estimation accuracy and computational costs. This is also true for the number M of randomly drawn control steps. A possible strategy would be to start with a moderate number M (or grid resolution) and increase this number (resolution) until the results stabilize, i.e. the estimated parameters hardly change anymore (Warton and Shepherd, 2010).

S2.2 Movement parameters and corresponding movement covariates

Depending on the assumptions made about the step length and turning angle distributions, different movement covariates must be included in analysis (Avgar et al., 2016; Nicosia et al., 2017). The meaning of the corresponding HMM-iSSA movement coefficients is related to the parameters of these distributions, e.g. the shape and rate parameters of the gamma distribution, but can slightly differ depending on the sampling procedure used to draw the control locations for the case-control data set. An overview for common step length distributions and the von Mises turning angle distribution is provided in Table S1. An overview of how to derive the natural distributional parameters, e.g. the shape and rate of the gamma distribution, from the corresponding HMM-iSSA movement coefficients is given in Table S2.

			coefficient interpretation		
distribution	covariate	coefficient	"importance" sampling	uniform sampling	grid approach
$\text{Exp}(\lambda_i)$	$-l$	$\theta_{i,-l}$	$\lambda_i - \lambda_{\text{prop}}$	λ_i	$\lambda_i^{(*)}$
$\text{Gamma}(k_i, r_i)$	$\log(l)$	$\theta_{i,\log(l)}$	$k_i - k_{\text{prop}}$	$k_i - 1$	$k_i - 2$
	$-l$	$\theta_{i,-l}$	$r_i - r_{\text{prop}}$	r_i	r_i
$\text{LN}(\mu_i, \sigma_i)$	$\log(l)$	$\theta_{i,\log(l)}$	$\frac{\mu_i}{\sigma_i^2} - \frac{\mu_{\text{prop}}}{\sigma_{\text{prop}}^2}$	$\frac{\mu_i}{\sigma_i^2} - 1$	$\frac{\mu_i}{\sigma_i^2} - 2$
	$-\log(l)^2$	$\theta_{i,-\log(l)^2}$	$\frac{1}{2\sigma_i^2} - \frac{1}{2\sigma_{\text{prop}}^2}$	$\frac{1}{2\sigma_i^2}$	$\frac{1}{2\sigma_i^2}$
$\text{vM}(0, \kappa_i)^{(**)}$	$\cos(\alpha)$	$\theta_{i,\cos(\alpha)}$	$\kappa_i - \kappa_{\text{prop}}$	κ_i	κ_i

Table S1: Example distributions for step length and turning angle. The table displays the associated movement covariates required for the Markov-switching conditional logistic regression, and the meaning of the corresponding movement coefficients for the three sampling procedures to sample the control locations as described in Section S2.1. The table provides information for the exponential distribution (Exp) with rate λ_i, Gamma distribution with shape k_i and rate r_i, log-normal (LN) distribution with mean μ_i and variance σ_i^2, and von Mises (vM) distribution with mean zero and concentration κ_i. (*) For the exponential distribution, $-\log(l)$ must be included as an offset in the grid approach. (**) Here, a negative estimate for κ_i can be interpreted as the concentration parameter of the von Mises distribution with mean π, i.e. $\text{vM}(\pi, \kappa_i)$

distribution	parameter	"importance" sampling	uniform sampling	grid approach
$\text{Exp}(\lambda_i)$	rate λ_i	$\theta_{i,-l} + \lambda_{\text{prop}}$	$\theta_{i,-l}$	$\theta_{i,-l}$
$\text{Gamma}(k_i, r_i)$	shape k_i	$\theta_{i,\log(l)} + k_{\text{prop}}$	$\theta_{i,\log(l)} + 1$	$\theta_{i,\log(l)} + 2$
	rate r_i	$\theta_{i,-l} + r_{\text{prop}}$	$\theta_{i,-l}$	$\theta_{i,-l}$
$\text{LN}(\mu_i, \sigma_i)$	mean μ_i	$\dfrac{\theta_{i,\log(l)} + \frac{\mu_{\text{prop}}}{\sigma^2_{\text{prop}}}}{2\left(\theta_{i,-\log(l)^2} + \frac{1}{2\sigma^2_{\text{prop}}}\right)}$	$\dfrac{\theta_{i,\log(l)} + 1}{2\theta_{i,-\log(l)^2}}$	$\dfrac{\theta_{i,\log(l)} + 2}{2\theta_{i,-\log(l)^2}}$
	variance σ_i^2	$\dfrac{1}{2}\left(\theta_{i,-\log(l)^2} + \frac{1}{2\sigma^2_{\text{prop}}}\right)^{-1}$	$\dfrac{1}{2\theta_{i,-\log(l)^2}}$	$\dfrac{1}{2\theta_{i,-\log(l)^2}}$
$\text{vM}(0, \kappa_i)^{(**)}$	concentration κ_i	$\theta_{i,\cos(\alpha)} + \kappa_{\text{prop}}$	$\theta_{i,\cos(\alpha)}$	$\theta_{i,\cos(\alpha)}$

Table S2: Overview of deriving the natural step length and turning angle parameters from the HMM-iSSA movement coefficients for the three control step sampling procedures described in Section S2.1. (**) Here, a negative estimate for κ_i can be interpreted as the concentration parameter of the von Mises distribution with mean π, i.e. $\text{vM}(\pi, \kappa_i)$

S3 Initial values for the maximum likelihood estimation

The HMM-iSSA requires starting values, i.e. first guesses, for each model parameter to initialize the parameter estimation. For each state $i = 1, \ldots, N$ the model comprises:

1. the state transition probabilities $\gamma_{ij} = \Pr(S_t = j \mid S_{t-1} = i)$ with $j = 1, \ldots, N$:
   - probabilities for the underlying state sequence to switch from state i to state $j = 1, \ldots, N$
   - values lie between zero and one, i.e. $0 \leq \gamma_{ij} \leq 1$
   - values must sum to one, i.e. $\sum_{j=1}^{N} \gamma_{ij} = 1$
   - usually, a certain persistence in the states is assumed, such that the probability to remain in the current state is rather high, e.g. values between 0.8 and 0.95
2. the initial state probability $\delta_i = \Pr(S_{t_0} = i)$:
   - probability for the state sequence to start in state i
   - values lie between zero and one, i.e. $0 \leq \delta_i \leq 1$
   - if stationarity is assumed for the Markov chain, the initial probabilities are not estimated but computed from the model's transition probability matrix
3. the state-dependent movement parameter vector $\boldsymbol{\theta}_i$:
   - contains the parameters of the movement kernel
   - depends on the distributions assumed for step length and turning angle (see Table S1)
   - for gamma distributed step length and von-Mises distributed turning angles, it comprises the shape k_i and rate r_i of the gamma distribution and the concentration parameter κ_i of the von Mises distribution; all values must be greater than zero
4. the state-dependent selection coefficient vector $\boldsymbol{\beta}_i$
   - contains the parameters for the selection function
   - comprises the selection coefficients for each habitat covariate

The log-likelihood (LL) function of the hidden Markov step selection model is usually multi-modal. Thus, it is possible that the optimization procedure ends up in a local maximum instead of the global maximum of the log-likelihood (MLL) required for the maximum likelihood estimates (MLEs). As the starting values build the starting point for the search of the function's maximum, they can have a large impact on the output of the optimization algorithm. Therefore, its is necessary to run the HMM-iSSA with different sets of (random) starting values to increase the chance of finding the MLEs. Among the set of HMM-iSSAs fitted with different starting values, the one with the largest corresponding MLL value provides the final results. However, to ensure numerical stability and convergence, it is necessary to check if different sets of initial values have lead to the same MLL value and MLEs. This can be done, for example, by plotting the MLL values found across the different initial values and by comparing the estimates of HMM-iSSAs with similar MLL values.

It can be inefficient to choose the starting values completely at random which can result in implausible values and in turn lead to poor model results (e.g. singular models). Therefore, it is usually useful to restrict the initial parameter values to plausible ranges and to ensure that the initial parameter values

differ between the N states. An inspection of the observed step length and turning angle observations, e.g. plotting the histograms and the raw data, might be helpful to find such plausible ranges.

In the following, we describe a possible procedure for selecting the starting values, which can serve as a starting point and can be adapted depending on the data and model at hand:

1. Choose large random values for the probabilities to remain in a given state (γ_{ii}), e.g. draws from a uniform distribution between 0.80 and 0.95 ($runif(N, 0.8, 0.95)$ in R).

   For the remaining transition probabilities, $\gamma_{ij} = \frac{1-\gamma_{ii}}{N-1}$ can be used for $i \neq j$. Otherwise, when drawing random starting values for the off-diagonals of the transition probability matrix, make sure that transition probabilities from state i to any other state sum to 1.

2. If no stationarity is assumed for the underlying latent Markov chain, the starting values for the initial state distribution can be fixed to $\delta_i = \frac{1}{N}$ or randomly drawn from a uniform distribution with values between 0 and 1. For the latter, the values must then be normalized with $\frac{\delta_i}{\sum_{i=1}^{N} \delta_i}$.

3. For the movement kernel, initial parameters for the step length distribution and possibly for the turning angle distribution are required.

   - Step length distribution: the quantiles of the step length observations can be used to draw random mean step length values μ_i (*quantile*-function in R). To avoid a large overlap between the mean values across the states, $N+1$ step length quantiles $q_1, \ldots, q_{N+1}$ can be considered for N states, for example with q_1 being the 0.1-quantile and q_{N+1} being the 0.9-quantile. The mean step length value μ_i for state i is then drawn from a uniform-distribution with values between q_i and q_{i+1}. The corresponding standard deviation σ_i can be drawn from a uniform distribution, for example, with values between a quarter and twice the mean value, i.e. $\frac{1}{4}\mu_i$ and $2\mu_i$. Based on these state-dependent mean values and standard deviations, the corresponding step length distribution parameters can be derived. For a gamma distribution, this is the shape with $k_i = \frac{\mu_i^2}{\sigma_i^2}$ and rate with $r_i = \frac{\mu_i}{\sigma_i^2}$.
   - Turning angles: The concentration parameter of the von Mises distribution can be randomly drawn from a uniform distribution with values between 0.2 and 2.

4. For the selection coefficients, draw random values from a set of plausible values, e.g. $\{-2.0, -1.0, -0.5, 0.0, 0.5, 1.0, 2.0\}$ (using the *sample*-function in R).

5. repeat the procedure several, e.g. 50 times.

As a starting point, our R-package *HMMiSSA* provides a function named *generate_starting_values* to draw random starting values for the HMM-iSSA.

S4 Supplementary material for the simulation study

S4.1 Simulated landscape feature

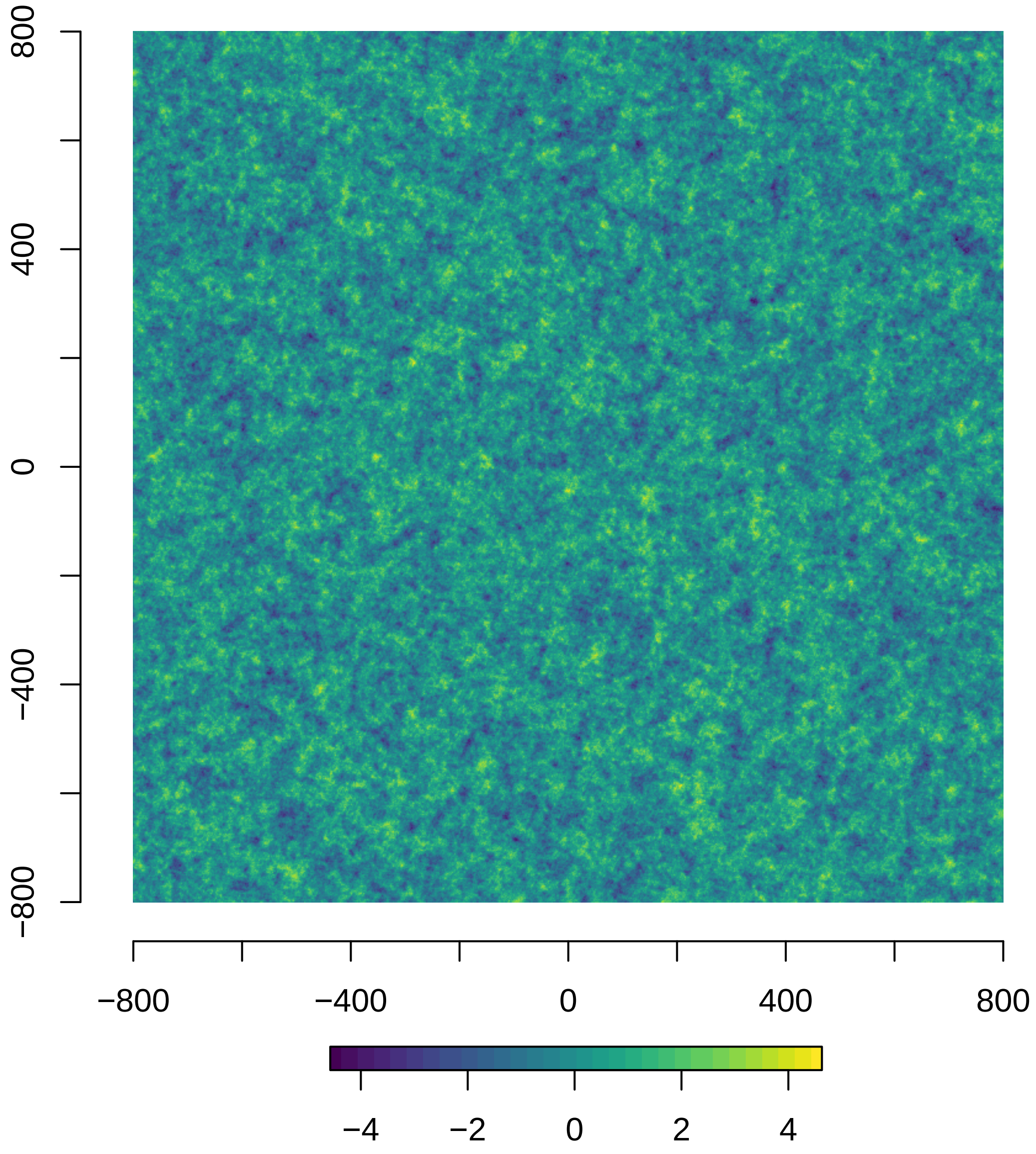

Figure S2: Simulated landscape feature map used to derive the habitat covariates in the simulation study described in Section 2.4 (main manuscript).

S4.2 Bias

scenario	method	no. cont.	select. coeff. β_1	select. coeff. β_2	shape k_1	shape k_2	rate r_1	rate r_2	conc. κ_1	conc. κ_2
1	iSSA	20	0.98	-1.02	-0.35	-1.65	-1.06	-0.10	0.18	-0.52
		100	1.00	-1.00	-0.35	-1.65	-1.07	-0.10	0.19	-0.51
		500	1.01	-0.99	-0.35	-1.65	-1.07	-0.11	0.19	-0.51
	TS-iSSA	20	0.19	-0.16	0.04	0.01	0.02	0.01	0.02	-0.07
		100	0.18	-0.14	0.04	0.02	0.02	0.01	0.03	-0.06
		500	0.18	-0.13	0.04	0.02	0.02	0.01	0.02	-0.06
	HMM-iSSA	20	0.01	-0.01	0.01	-0.07	0.03	-0.01	0.00	-0.02
		100	0.02	-0.01	0.02	0.00	0.02	0.00	0.00	0.00
		500	0.02	-0.01	0.02	0.03	0.02	0.00	0.00	0.00
2	iSSA	20	1.99	-2.01	-0.76	-0.76	-0.06	-0.06	-0.30	-0.30
		100	1.99	-2.01	-0.76	-0.76	-0.06	-0.06	-0.30	-0.30
		500	1.99	-2.01	-0.76	-0.76	-0.06	-0.06	-0.30	-0.30
	TS-iSSA	20	1.96	-1.97	-0.76	-0.76	-0.06	-0.06	-0.30	-0.30
		100	1.96	-1.97	-0.76	-0.75	-0.06	-0.06	-0.30	-0.30
		500	1.96	-1.97	-0.76	-0.75	-0.06	-0.06	-0.30	-0.30
	HMM-iSSA	20	-0.02	0.01	-0.01	-0.03	0.00	0.00	0.00	0.00
		100	-0.01	0.00	-0.01	-0.03	0.00	0.00	0.00	0.00
		500	0.00	-0.01	-0.01	-0.03	0.00	0.00	-0.02	-0.02
3	iSSA	20	-0.01	-0.01	-0.50	-1.80	-1.10	-0.14	0.32	-0.38
		100	-0.01	-0.01	-0.49	-1.79	-1.10	-0.14	0.32	-0.38
		500	-0.01	-0.01	-0.49	-1.79	-1.10	-0.14	0.32	-0.38
	TS-iSSA	20	0.00	0.00	0.03	0.12	0.04	0.01	0.00	0.01
		100	0.00	0.00	0.03	0.12	0.04	0.01	0.00	0.01
		500	0.00	0.00	0.03	0.12	0.04	0.01	0.00	0.01
	HMM-iSSA	20	0.00	0.00	0.01	-0.02	0.01	0.00	0.00	0.00
		100	0.00	0.00	0.01	0.01	0.01	0.00	0.00	0.00
		500	0.00	0.00	0.02	0.02	0.01	0.00	0.00	0.00

Table S3: Estimation bias for the model parameters for each simulation scenario and applied method. The bias is calculated as the mean difference between the parameter estimate and the true parameter value across the 100 simulation runs.

S4.3 Root mean squared error

			select. coeff.		shape		rate		conc.	
scenario	model	no. cont.	β_1	β_2	k_1	k_2	r_1	r_2	κ_1	κ_2
1	iSSA	20	0.98	1.03	0.36	1.65	1.06	0.10	0.19	0.52
		100	1.01	1.01	0.36	1.65	1.07	0.11	0.20	0.52
		500	1.01	1.00	0.36	1.66	1.07	0.11	0.20	0.52
	TS-iSSA	20	0.23	0.18	0.08	0.23	0.13	0.04	0.08	0.13
		100	0.23	0.16	0.08	0.21	0.13	0.04	0.07	0.11
		500	0.22	0.15	0.08	0.21	0.13	0.03	0.07	0.11
	HMM-iSSA	20	0.12	0.10	0.07	0.20	0.12	0.04	0.07	0.11
		100	0.12	0.08	0.07	0.17	0.11	0.03	0.07	0.09
		500	0.12	0.07	0.07	0.17	0.10	0.03	0.07	0.09
2	iSSA	20	1.99	2.01	0.77	0.77	0.07	0.07	0.31	0.31
		100	1.99	2.02	0.76	0.76	0.07	0.07	0.31	0.31
		500	1.99	2.02	0.76	0.76	0.07	0.07	0.31	0.31
	TS-iSSA	20	1.99	2.01	0.77	0.77	0.07	0.07	0.31	0.31
		100	1.99	2.02	0.76	0.76	0.07	0.07	0.31	0.31
		500	1.99	2.02	0.76	0.76	0.07	0.07	0.31	0.31
	HMM-iSSA	20	0.09	0.09	0.18	0.17	0.02	0.02	0.10	0.10
		100	0.08	0.08	0.17	0.17	0.02	0.02	0.09	0.09
		500	0.08	0.08	0.16	0.17	0.02	0.02	0.16	0.17
3	iSSA	20	0.07	0.07	0.50	1.80	1.10	0.14	0.33	0.38
		100	0.07	0.07	0.50	1.80	1.10	0.14	0.33	0.38
		500	0.07	0.07	0.50	1.80	1.10	0.14	0.33	0.38
	TS-iSSA	20	0.12	0.06	0.08	0.26	0.11	0.03	0.07	0.09
		100	0.12	0.06	0.08	0.26	0.11	0.03	0.06	0.09
		500	0.12	0.06	0.08	0.26	0.11	0.03	0.06	0.09
	HMM-iSSA	20	0.13	0.07	0.07	0.22	0.10	0.02	0.07	0.10
		100	0.12	0.06	0.07	0.21	0.10	0.02	0.07	0.09
		500	0.12	0.06	0.07	0.21	0.10	0.02	0.07	0.09

Table S4: Root mean squared error of the estimated parameters calculated across the 100 simulation runs for each fitted model and simulation scenario, respectively. The root mean squared error is calculated as the square root of the average squared difference between the parameter estimate and the true parameter value across the 100 simulation runs.

S5 Additional simulation runs

S5.1 Uniform sampling of the control locations

We re-ran the simulation study described in Section 2.4 (main manuscript) with uniformly sampled random steps for the HMM-iSSA. The results are comparable to the results described in the main manuscript (Figure S3). However, with uniform sampling, the results seem to be more sensitive to the number M of control steps used for each observed step. For example, with $M = 20$ control locations, the rate parameters in Scenario 1 and 2 are slightly biased. Thus, $M = 20$ control locations might be insufficient here.

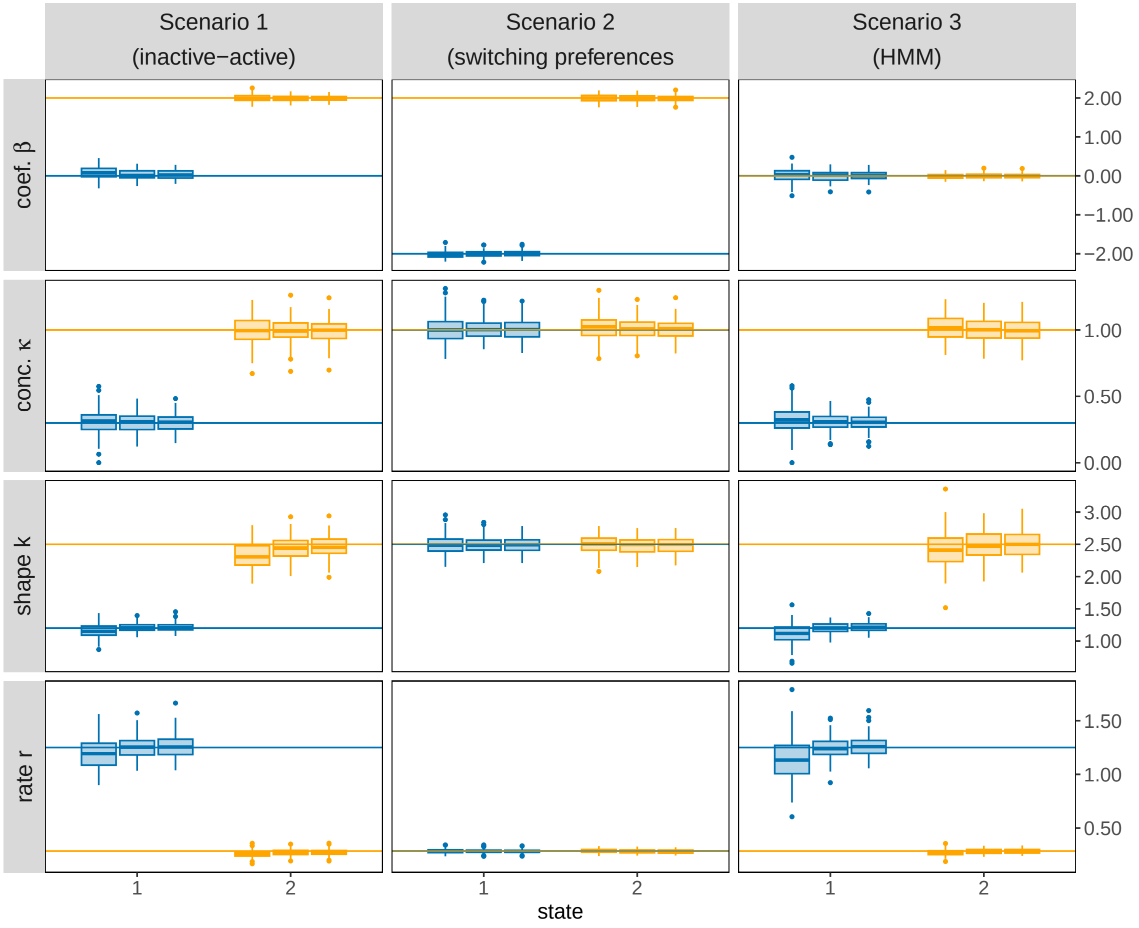

Figure S3: Uniform sampling of the control locations: Boxplots of the HMM-iSSA parameter estimates across the 100 simulation runs for each simulation scenario and number of control locations M, respectively. The control locations for the case-control locations were derived from uniformly sampled step lengths and turning angles. Here, for the upper step length limit, 10 was added to the maximum observed step length. The rows refer to the estimated selection coefficient (beta), the shape and rate of the gamma distribution for step length and the concentration parameter (kappa) of the von Mises distribution for turning angle, respectively. The columns refer to the three different simulation scenarios. The colors indicate the different states (state 1: blue, state 2: orange), the three adjacent boxplots refer the use of $M = 20$, $M = 100$ and $M = 500$ control locations per used location for the parameter estimation.

S5.2 Scenario without state-switching

To check the robustness of the HMM-iSSA for data without underlying behavioral states, we ran a fourth simulation scenario with data generated from a standard iSSA. For the movement-kernel, we used gamma distributed step length with shape $k = 2.5$ and rate $r = 0.29$ and von-Mises distributed turning angles with mean zero and concentration $\kappa = 1$. The selection coefficient was set to $\beta = 2$. A corresponding 2-state HMM-iSSA would need to share the same movement and selection coefficients across both states (see Section 2.1, main manuscript). However, as state 1 and 2 do not differ, a switch from one state to the other can occur arbitrarily. Therefore, the transition probability matrix is not identified in this scenario and could take any form. This could lead to numerical problems and instabilities in the HMM-iSSA parameter estimation.

To initialize the parameter estimation, we used the true parameter values and 50 sets of randomly drawn parameter values as starting values. With the true values being the starting values, the estimated parameters did not diverge and the HMM-iSSA performed well (Figure S4). This was, however, not the case when testing 50 sets of random starting values (Figure S5). While the estimates corresponding to state 1 of the 2-state HMM-iSSA seem fine, the estimates of state 2 completely diverged. This was especially the case for the shape and rate parameter of the gamma distribution.

This highlights the importance to carefully inspect the estimated HMM-iSSA before interpreting the results. For this simulation scenario, there are several indications that the applied 2-state HMM-iSSAs are not appropriate for the simulated data sets, for example:

1. Maximum likelihood values found across the 50 sets of random starting values:

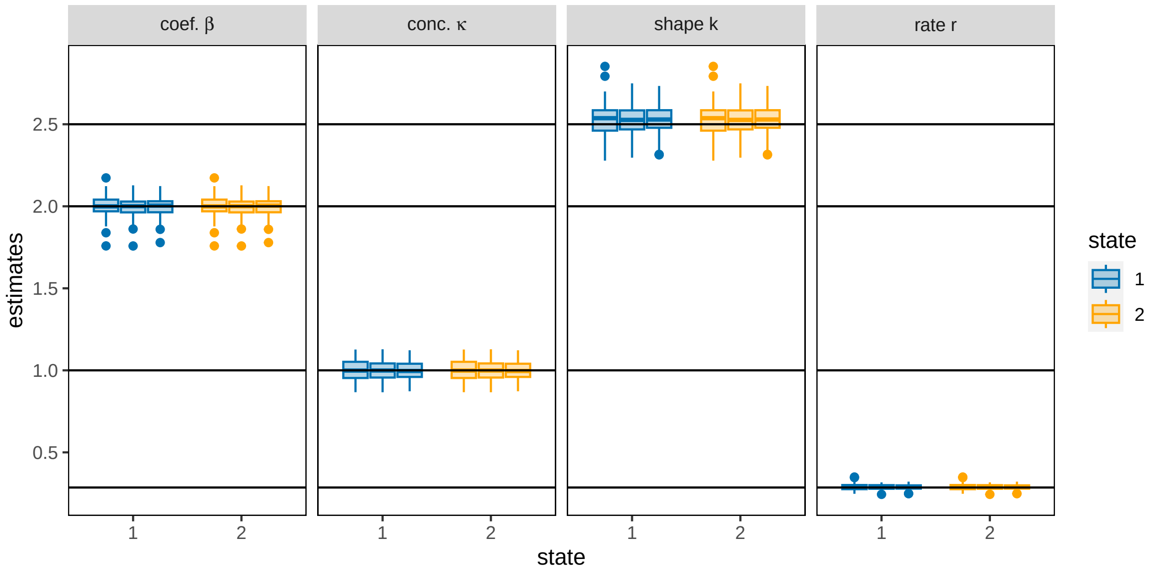

Figure S4: Boxplots of the HMM-iSSA parameter estimates across the 100 simulation runs for the iSSA-scenario for each number of control locations M, respectively. Here, the true values were used as starting values for the Markov-switching conditional logistic regression. The figure columns refer to the estimated selection coefficient (beta), the shape and rate of the gamma distribution for step length and the concentration parameter (kappa) of the von Mises distribution for turning angle, respectively. The colors indicate the different states (state 1: blue, state 2: orange), the three adjacent boxplots refer the use of $M = 20$, $M = 100$ and $M = 500$ control locations per used location for the parameter estimation.

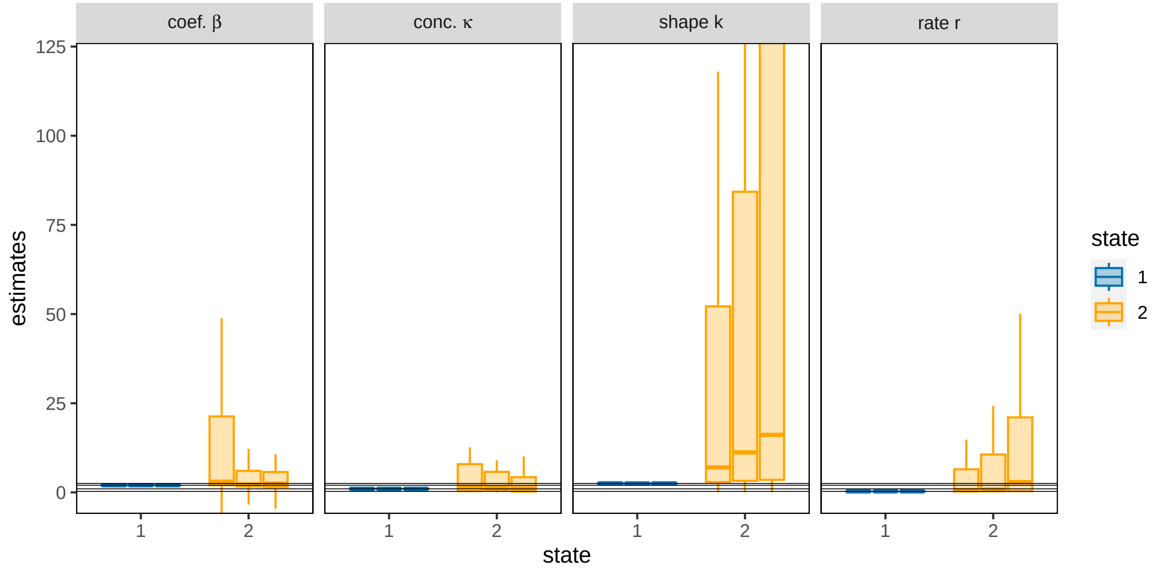

Figure S5: Boxplots of the HMM-iSSA parameter estimates across the 100 simulation runs for the iSSA-scenario for each number of control locations M, respectively. Here, 50 random starting values were used as starting values for the Markov-switching conditional logistic regression. The figure columns refer to the estimated selection coefficient (beta), the shape and rate of the gamma distribution for step length and the concentration parameter (kappa) of the von Mises distribution for turning angle, respectively. The colors indicate the different states (state 1: blue, state 2: orange), the three adjacent boxplots refer the use of $M = 20$, $M = 100$ and $M = 500$ control locations per used location for the parameter estimation.

In some simulation runs only a single set of initial values led to the maximum likelihood value found, the remaining 49 sets of initial values led to smaller log-likelihood values. This usually indicates numerical instability and problems in the estimated model.

2. Estimated transition probabilities and Viterbi decoding:

   In some simulation runs the movement and selection coefficients of state 2 differed between HMM-iSSAs with similar log-likelihood values, i.e. different sets of starting values resulted in similar log-likelihood values, but different estimates for state 2. This corresponds to problems in the estimated transition probability matrix, namely that the probability to stay in state 1, γ_{11}, is (almost) equal to one, while the probability to stay in state 2, γ_{22}, is low (Figure S6). Consequently, it is possible that state 2 is rarely or even never visited. This is also indicated by the Viterbi-decoded state sequences, which assigned less than 1% of the data to state 2 in 49% ($M = 20$), 46% ($M = 100$) and 58% ($M = 500$) of the simulation runs, respectively. Furthermore, in some simulation runs the probability to remain in the current state was low for both states, e.g. $\gamma_{11} < 0.2$ for all states $i = 1, \ldots, N$. This would correspond to a permanent switching between the states which is usually implausible in the context of animal movement data.

3. Estimated movement parameters:

   The shape, rate and concentration parameters must be greater than zero. In some simulation runs, however, they were estimated to be (almost) zero, which corresponds to their boundary values and indicates serious model problems.

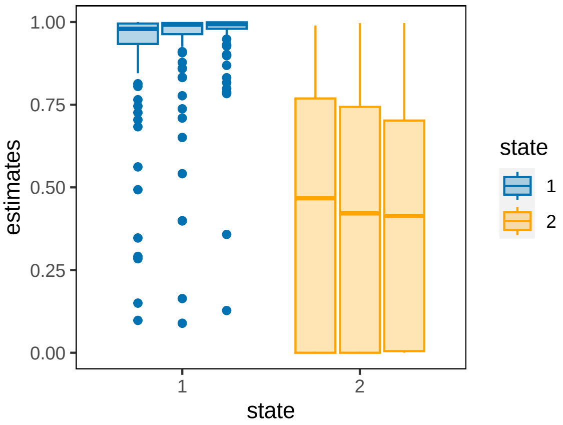

Figure S6: Boxplots of the estimated HMM-iSSA transition probabilities γ_{11} and γ_{22}, i.e. the probabilities to remain in the same state, across the 100 simulation runs for the iSSA-scenario for each number of control locations M, respectively. Here, 50 random starting values were used as starting values for the Markov-switching conditional logistic regression. The colors indicate the different states (state 1: blue, state 2: orange), the three adjacent boxplots refer the use of $M = 20$, $M = 100$ and $M = 500$ control locations per used location for the parameter estimation.

4. Variance of the state-dependent gamma distribution for step length:

   In some simulation runs, the variance of the state-dependent gamma distribution for state 2, as derived from the estimated shape and rate parameters, was very low (below 0.1). This implies that state 2 only covers very specific step length values.

Usually, several of such problems occur together. Furthermore, in this simulation scenario, the AIC performed worse in selecting the correct model (Table S5). Thus, this simulation scenario highlights the importance to test several sets of starting values to derive the maximum likelihood estimates and to closely inspect the model results.

	AIC		BIC	
no. cont.	iSSA	HMM-iSSA	iSSA	HMM-iSSA
20	**53**	47	**100**	0
100	**50**	50	**100**	0
500	**53**	47	**100**	0

Table S5: Percentage of simulation runs in which either the iSSA or the HMM-iSSA was selected by either AIC or BIC for each number of control steps used for model fitting, respectively. The cells belonging to the true underlying model are highlighted in bold face.

S5.3 Habitat feature with less spatial variation

We re-ran the simulation study (Section 2.4, main manuscript) again using a landscape feature map with less spatial variation, i.e. a more homogeneous landscape (Figure S7). The results are similar to the results of our original simulation study, but the variance in the parameter estimates increased (Figure S8). Moreover, the classification performance of the HMM improves especially in Scenario 1 as with less spatial variation in the habitat features, the influence of the movement kernel on the movement track increases (Table S6). This also leads to a decline in the classification performance of the HMM-iSSA in Scenario 2.

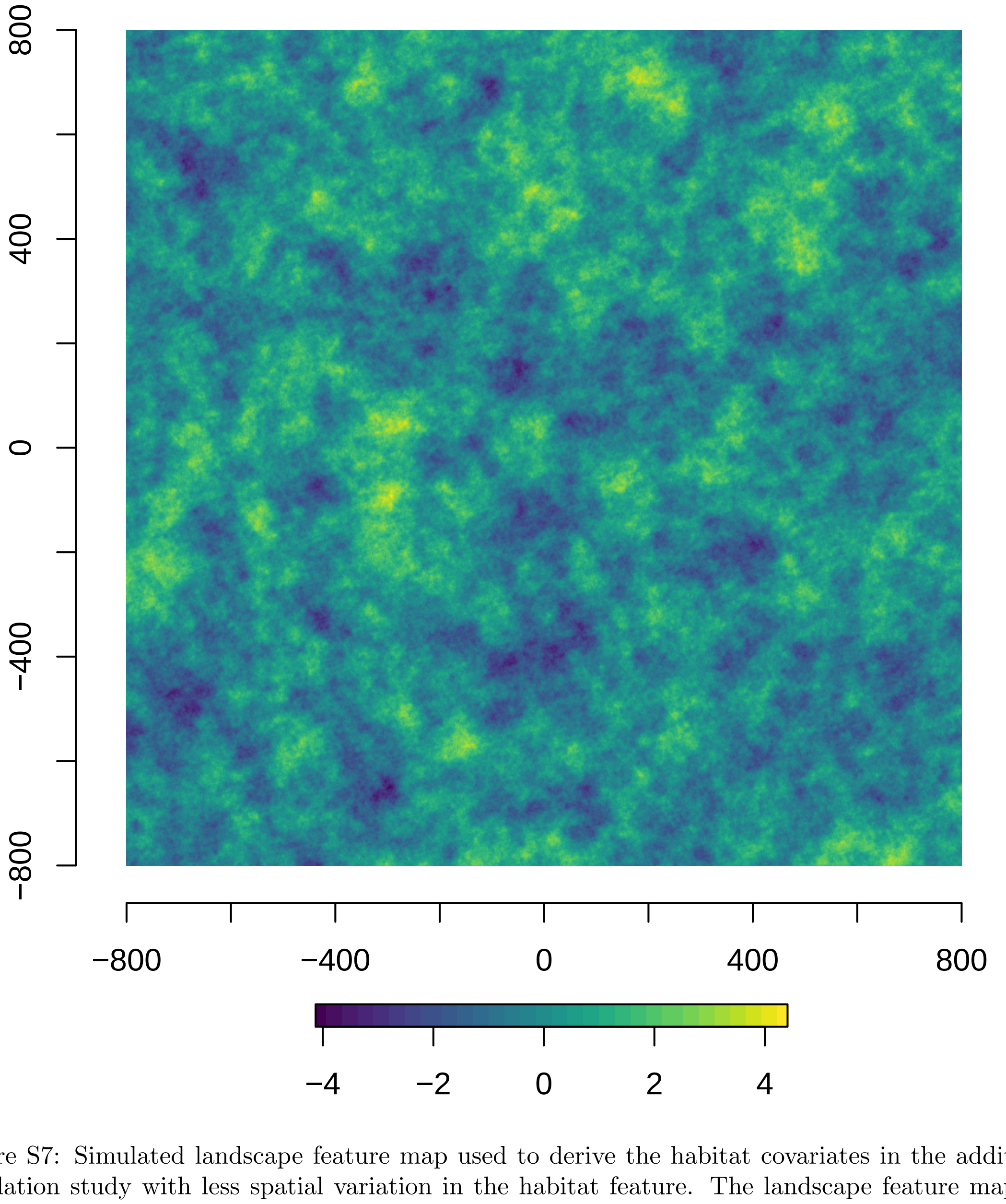

Figure S7: Simulated landscape feature map used to derive the habitat covariates in the additional simulation study with less spatial variation in the habitat feature. The landscape feature map is a realization of a Gaussian random field with covariance 1 and range 50.

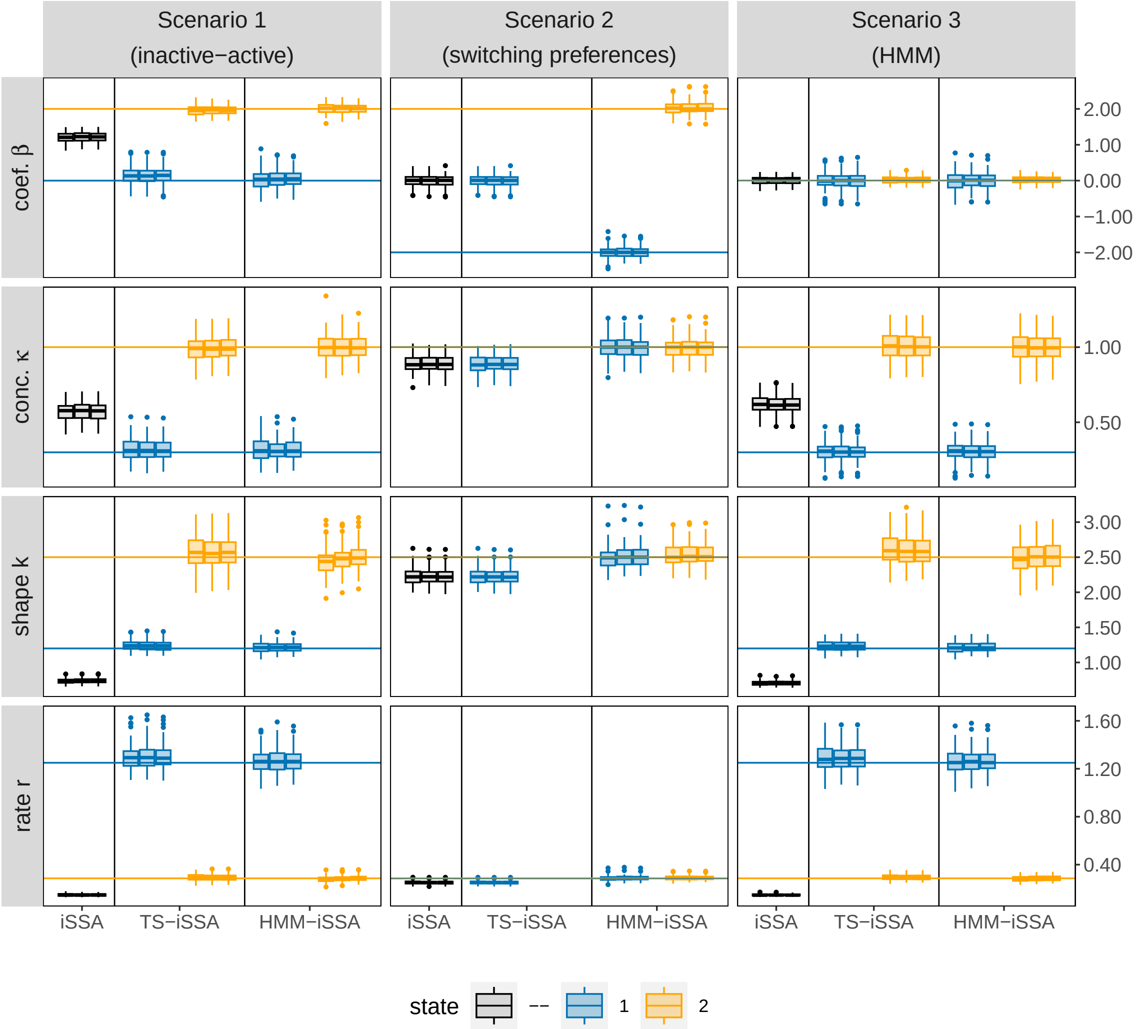

Figure S8: Boxplots of the parameter estimates across the 100 simulation runs in the simulation run with less spatial variation in the landscape feature. Results are shown for each applied method, simulation scenario and number of control locations M, respectively. The rows refer to the estimated selection coefficient (beta), the shape and rate of the gamma distribution for step length and the concentration parameter (kappa) of the von Mises distribution for turning angle, respectively. The columns refer to the three different simulation scenarios. For each method (iSSA, TS-iSSA and HMM-iSSA) and state (state 1: blue, state 2: orange, no state differentiation: black), the three adjacent boxplots refer the use of $M = 20$, $M = 100$ and $M = 500$ control locations per used location for the parameter estimation. Note that in Scenario 2, the TS-iSSA is naturally not capable to distinguish between two states as both share the same movement kernel. Thus, there are only results for a single state.

	HMM-iSSA 20		HMM-iSSA 100		HMM-iSSA 500		HMM	
scen. 1	2.83	(0.68)	2.73	(0.68)	**2.70**	(0.64)	2.98	(0.65)
scen. 2	8.98	(1.67)	8.56	(1.64)	**8.55**	(1.78)	49.48	(4.46)
scen. 3	2.48	(0.49)	2.42	(0.51)	**2.37**	(0.53)	2.39	(0.53)

Table S6: Simulation runs with less spatial variation in the habitat feature: Mean missclassification rate with standard deviation in parentheses across the 100 simulation runs for each scenario and fitted state-switching model, respectively. The missclassification rate is calculated as the percentage of states incorrectly classified using the Viterbi sequence. The lowest missclassification rate for each scenario is highlighted in bold face.

S6 Supplementary material for the bank vole case study

S6.1 Animal care approval and research permissions

The research was following the guidelines of the Amercian Society of Mammologist for the use of wild mammals in research, including telemetry (Sikes and the Animal Care and Use Committee of the American Society of Mammalogists, 2016). It complied with legislation in Germany for animal experimentation (§8 of animal protection law (TschG), §33-43 Tierschutzversuchstierverordnung, §2 of Tierschuztzustänidgkeitsverordnung) with the permission by the Landesamt für Umwelt, Gesundheit und Verbraucherschutz Brandenburg (LUGV; reference number V3-2347-44-2011), legislation about wildlife capture (exemptions for the Nature protection law 43 Abs. 8 Nr. 3 Bundesnaturschutzgesetz (BNatSchG) and §72 Abs. 3 Brandenburgisches Naturschutzgesetz (BbgNatSchG)) with permission by Landesumweltamt Brandenburg (reference number RW7.1 24.01.01.10), and legislation about keeping animals (§11 Abs.1 Nr. 1 animal protection law (TierschG), permitted by the LUGV 386-1).

S6.2 Selection coefficient estimates for different numbers of control steps

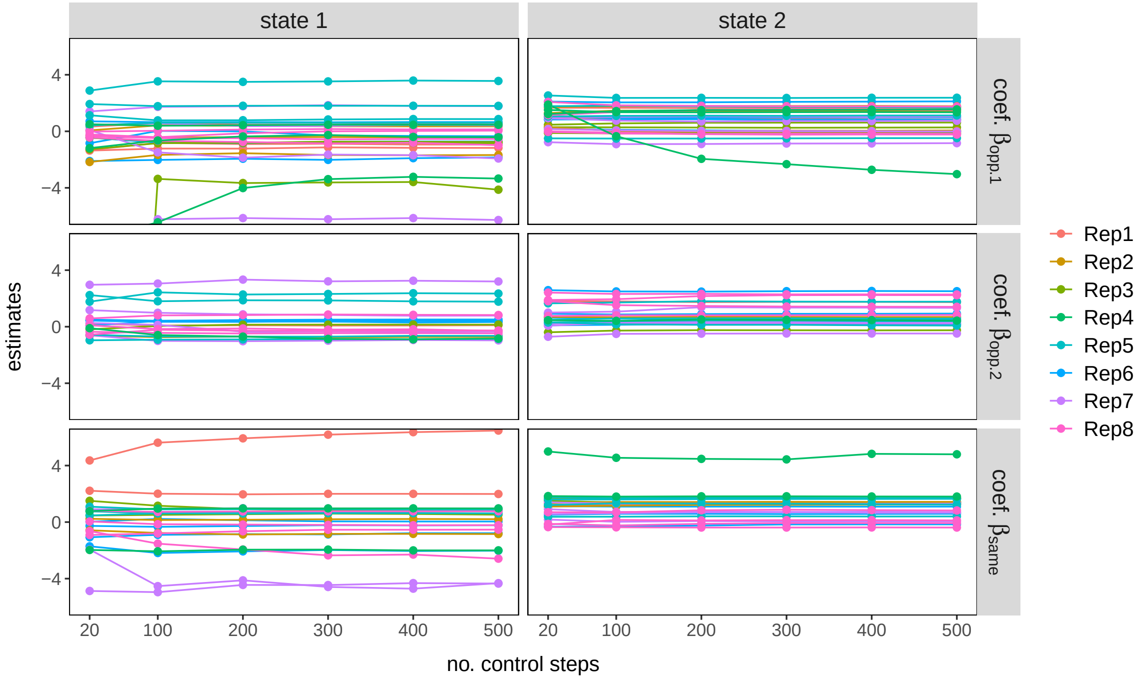

Figure S9: Estimated HMM-iSSA selection coefficients for opposite ($\beta_{opp.1}$, $\beta_{opp.2}$) and same sex (β_{same}) using different numbers of control steps M. With increasing M, all estimates stabilize. The state-dependent shape and rate estimates are also stable (results not shown).

S6.3 HMM-iSSA estimates for interactions with same-sex conspecifics

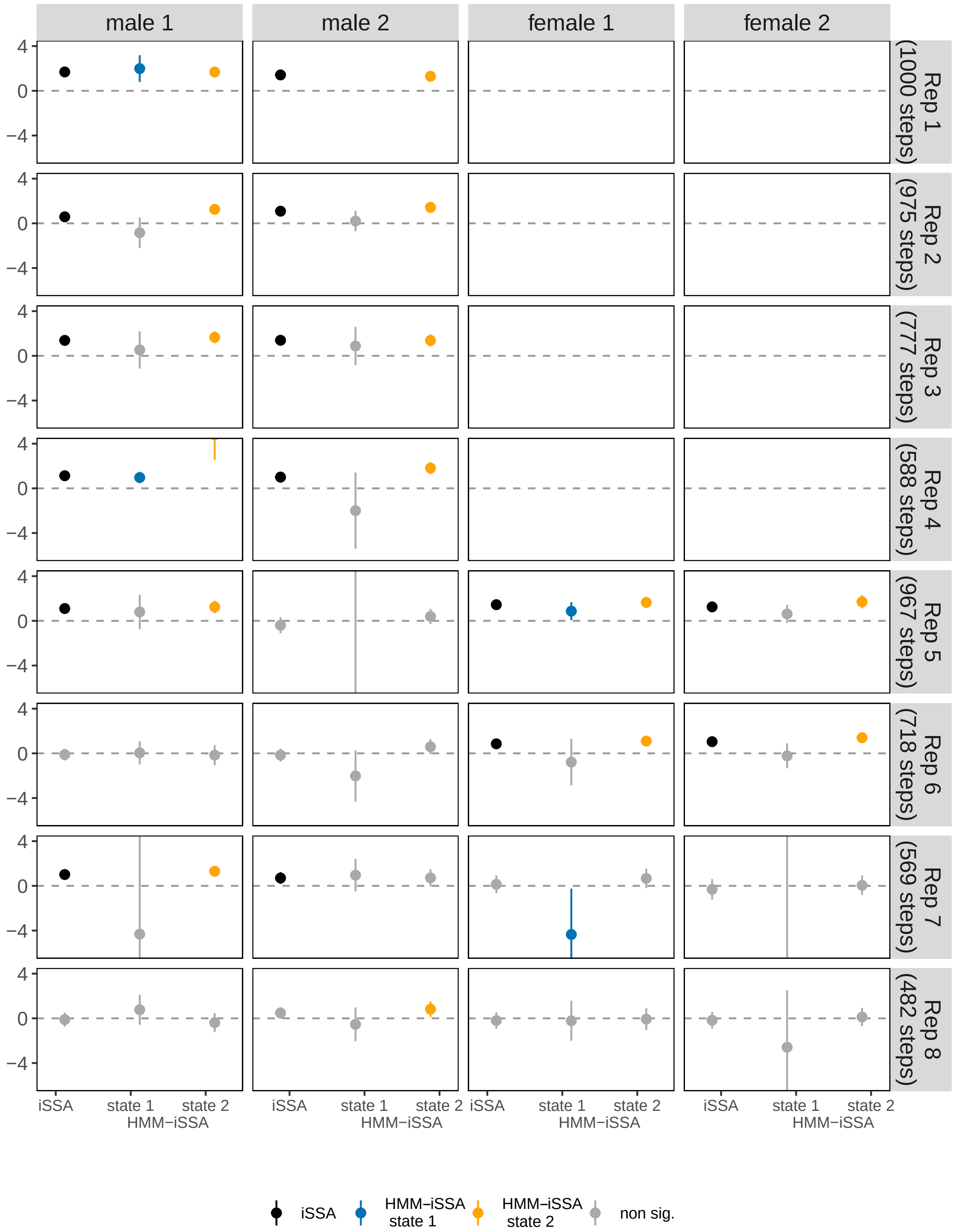

Figure S10: Estimated iSSA and HMM-iSSA selection coefficients (solid points/triangles) of interaction behavior between individuals of same sexes within the eight replicates (1–8), including 95% confidence intervals (solid lines). Each replicate consisted of two males (male 1 and male 2) and one or two females (female 1 and female 2) such that each individual could respond to one same-sex individual. Non-significant coefficients (p-values below 0.05) are grayed out. The horizontal dashed line indicates zero (i.e. neutral behavior); positive and negative coefficients indicate attraction and avoidance, respectively.

S6.4 TS-iSSA results

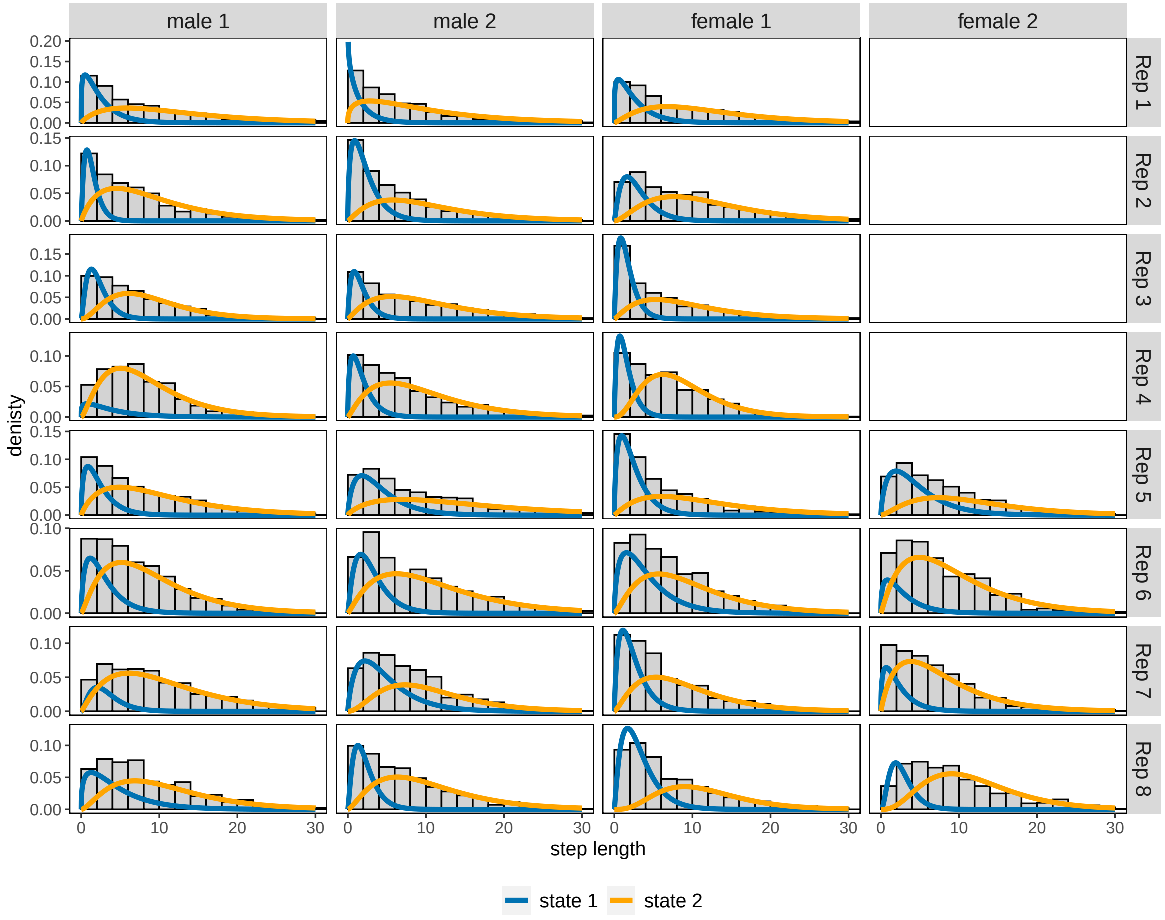

Figure S11: Estimated state-dependent gamma distributions for step length as implied by the fitted 2-state TS-iSSAs for each individual in replicates $1-8$, respectively. The distributions are weighted by the relative state occupancy frequencies derived from the Viterbi sequence. The gray histograms in the background show the distribution of the observed step length.

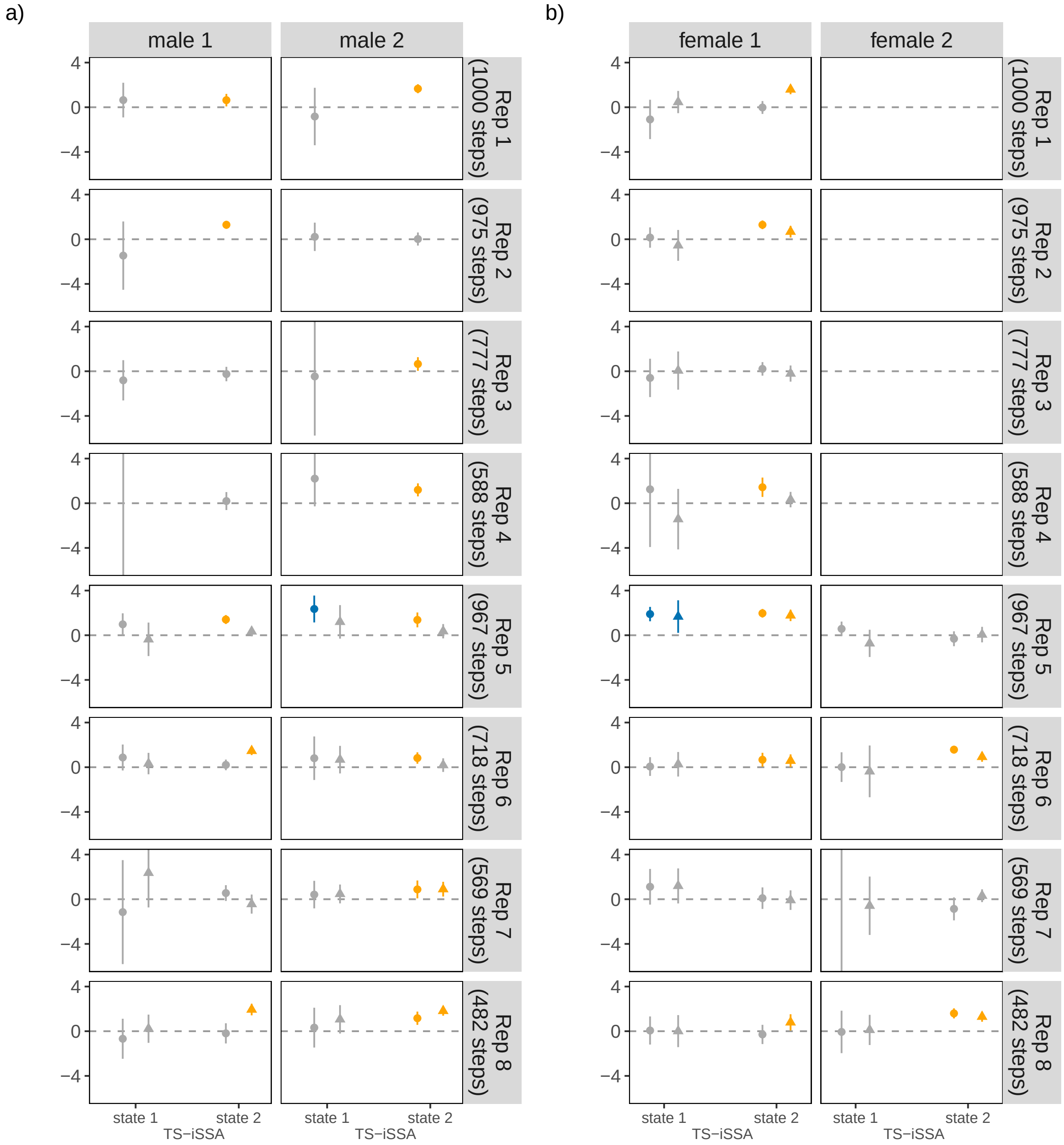

Figure S12: Estimated TS-iSSA selection coefficients (solid points/triangles) of interaction behavior between individuals of opposing sexes within the eight replicates (1–8), including 95% confidence intervals (solid lines). Each replicate consisted of two males (male 1 and male 2) and one or two females (female 1 and female 2) such that each individual could respond to up to two opposite-sex individuals (dot: response to female/male 1, triangle: response to female/male 2 within a replicate). Non-significant coefficients (p-values below 0.05) are grayed out. The horizontal dashed line indicates zero (i.e. neutral behavior); positive coefficients indicate attraction, while negative coefficients would indicate avoidance

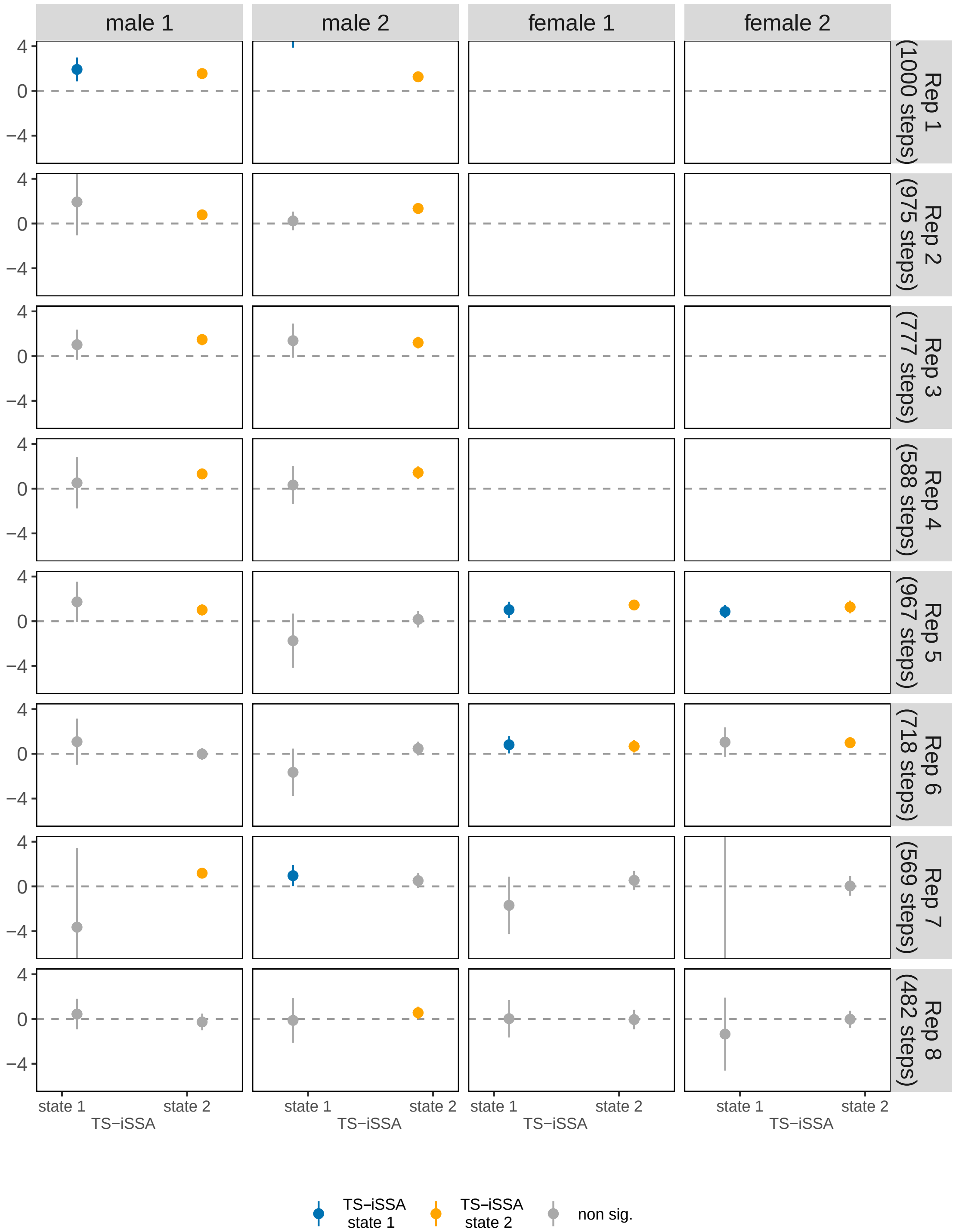

Figure S13: Estimated TS-iSSA selection coefficients (solid points/triangles) of interaction behavior between individuals of same sexes within the eight replicates (1–8), including 95% confidence intervals (solid lines). Each replicate consisted of two males (male 1 and male 2) and one or two females (female 1 and female 2) such that each individual could respond to one same-sex individual. Non-significant coefficients (p-values below 0.05) are grayed out. The horizontal dashed line indicates zero (i.e. neutral behavior); positive coefficients indicate attraction, while negative coefficients would indicate avoidance.

S6.5 Viterbi-decoding

		HMM		HMM-iSSA	
Rep.	Ind.	State 1	State 2	State 1	State 2
1	male 1	37.60	62.40	38.20	61.80
	male 2	25.40	74.60	24.50	75.50
	female 1	36.60	63.40	37.90	62.10
2	male 1	24.41	75.59	43.00	56.10
	male 2	46.77	53.23	48.10	51.90
	female 1	32.41	67.59	31.90	68.10
3	male 1	33.72	66.28	33.98	66.02
	male 2	26.64	73.36	26.64	73.36
	female 1	42.86	57.14	42.86	57.14
4	male 1	11.22	88.78	96.43	3.57
	male 2	25.68	74.32	21.94	78.06
	female 1	28.23	71.77	33.67	66.33
5	male 1	28.54	71.46	38.16	61.84
	male 2	40.74	59.26	39.71	60.29
	female 1	47.57	52.43	49.22	50.58
	female 2	50.98	49.02	47.57	52.43
6	male 1	26.04	73.96	66.71	33.29
	male 2	29.11	70.89	33.29	66.71
	female 1	40.67	59.33	22.42	77.58
	female 2	15.18	84.82	32.87	67.13
7	male 1	14.76	85.24	15.29	84.71
	male 2	49.38	50.62	48.51	51.49
	female 1	39.89	60.11	37.43	62.57
	female 2	20.04	79.96	22.67	77.33
8	male 1	36.31	63.69	44.61	55.39
	male 2	30.08	69.92	46.06	53.94
	female 1	54.98	45.02	57.26	42.47
	female 2	24.27	75.73	24.69	75.31

Table S7: Percentage of observed steps that was decoded to belong to state 1 or state 2 according to the HMM (i.e. TS-iSSA) or HMM-iSSA Viterbi sequence for each of the 28 bank vole individuals.

S6.6 Information criteria results

Rep.	No. steps	Ind.	AIC	ΔAIC	BIC	ΔBIC
1	1000	male 1	HMM-iSSA	64.10	HMM-iSSA	44.46
		male 2	HMM-iSSA	120.51	HMM-iSSA	100.88
		female 1	HMM-iSSA	43.90	HMM-iSSA	24.27
2	975	male 1	HMM-iSSA	79.87	HMM-iSSA	60.34
		male 2	HMM-iSSA	28.94	HMM-iSSA	9.41
		female 1	HMM-iSSA	49.66	HMM-iSSA	30.13
3	777	male 1	HMM-iSSA	27.73	HMM-iSSA	9.11
		male 2	HMM-iSSA	23.31	HMM-iSSA	4.69
		female 1	HMM*	6.43	HMM*	25.05
4	588	male 1	HMM-iSSA	23.62	iSSA	7.02
		male 2	HMM-iSSA	47.20	HMM-iSSA	29.70
		female 1	HMM-iSSA	9.88	HMM*	7.63
5	967	male 1	HMM-iSSA	83.41	HMM-iSSA	54.17
		male 2	HMM-iSSA	47.21	HMM-iSSA	17.97
		female 1	HMM-iSSA	239.92	HMM-iSSA	210.68
		female 2	HMM-iSSA	28.75	HMM*	0.50
6	718	male 1	HMM-iSSA	74.52	HMM-iSSA	47.07
		male 2	HMM-iSSA	13.93	HMM*	13.53
		female 1	HMM-iSSA	31.99	HMM-iSSA	4.53
		female 2	HMM-iSSA	72.20	HMM-iSSA	35.59
7	569	male 1	HMM-iSSA	27.22	HMM-iSSA	1.16
		male 2	HMM-iSSA	7.41	HMM*	16.17
		female 1	HMM*	0.54	HMM*	26.61
		female 2	HMM-iSSA	0.37	HMM*	25.70
8	482	male 1	HMM-iSSA	46.04	HMM-iSSA	20.97
		male 2	HMM-iSSA	75.30	HMM-iSSA	41.88
		female 1	HMM*	7.41	HMM*	32.48
		female 2	HMM-iSSA	13.66	iSSA	19.76

Table S8: Models selected by either AIC or BIC for each of the 28 bank vole individuals. The set of candidate models included the iSSA model, HMM* (i.e. HMM-iSSA without selection covariates) and HMM-iSSA model. Δ AIC and Δ BIC show the differences in AIC and BIC values between the best and second best model in the ranking.

References

Arce Guillen, R., Lindgren, F., Muff, S., Glass, T. W., Breed, G. A., and Schlägel, U. E. (2023). Accounting for unobserved spatial variation in step selection analyses of animal movement via spatial random effects. *Methods in Ecology and Evolution*, 14(10):2639–2653.

Avgar, T., Potts, J. R., Lewis, M. A., and Boyce, M. S. (2016). Integrated step selection analysis: bridging the gap between resource selection and animal movement. *Methods in Ecology and Evolution*, 7(5):619–630.

Nicosia, A., Duchesne, T., Rivest, L.-P., and Fortin, D. (2017). A multi-state conditional logistic regression model for the analysis of animal movement. *The Annals of Applied Statistics*, 11(3):1537–1560.

Schlägel, U. E. and Lewis, M. A. (2014). Detecting effects of spatial memory and dynamic information on animal movement decisions. *Methods in Ecology and Evolution*, 5(11):1236–1246.

Sikes, R. S. and the Animal Care and Use Committee of the American Society of Mammalogists (2016). 2016 Guidelines of the American Society of Mammalogists for the use of wild mammals in research and education. *Journal of Mammalogy*, 97(3):663–688.

Warton, D. I. and Shepherd, L. C. (2010). Poisson point process models solve the "pseudo-absence problem" for presence-only data in ecology. *The Annals of Applied Statistics*, 4(3):1383–1402.
